# The effects of slope gradient and soil depth on soil physicochemical properties in the Achew small-scale irrigation area, Southwest Ethiopia

**DOI:** 10.1371/journal.pone.0333894

**Published:** 2025-10-21

**Authors:** Wasihun Mengiste, Dereje Tsegaye, Samuel Dagalo, Teshome Yitbareke

**Affiliations:** 1 Department of Plant Science, Arba Minch University, Arba Minch, Ethiopia; 2 Faculties of Water Resources and Irrigation Engineering, Arba Minch University, Arba Minch, Ethiopia; 3 Natural Resource Management Welkite University, Welkite, Ethiopia; Jimma University College of Agriculture and Veterinary Medicine, ETHIOPIA

## Abstract

For sustainable land management, clear information about landscapes and soil depth is crucial. The purpose of this study was to investigate how slope variation and soil depth affect selected physicochemical properties of soil in the Achewa irrigated area of the Itang Special District in the Gambella region southwest of part of Ethiopia. Soil samples were collected from three topography positions (lower, middle, and upper slope locations) and on both soil samplings (0–20 cm and 20–40 cm). A total of 18 composite soil samples were collected from three slope gradients and two soil depths with three replications by auger for soil physical and chemical properties. Correspondingly, 18 undisturbed samples were taken for soil BD and TP determination by the core sampling method. Standard laboratory methods were used to analyze the collected soil samples. The data were analyzed using the General Linear Model (GLM) procedure in SAS version 9.4. The result showed that slope gradients, soil depths, and slope gradients interacting with soil depths had very highly, highly and significant effects on selected soil parameters, exchangeable bases, and extractable micronutrients. The highest percentage of sand fraction (32.00%) and BD (1.37) were recorded on the upper slope gradients. Beside the highest percentage of clay content (58.17%), TP (51.32%), pH (7.28), OM (3.52%), TN (0.21%), AV.P (26 ppm), exchangeable Ca (8.55 mg/l), Na (0.69 mg/l), K (0.43 mg/l), CEC (28.96 cmol (+) kg ⁻ ¹), PBS (56.44%), extractable Fe (15.78 mg kg ⁻ ¹), Mn (11.08 mg kg ⁻ ¹), and Cu (2.82 mg kg-1) were observed on the lower slope gradient, while, except for the sand fraction, BD, and extractable Zn, which are lowest on the lower slope gradients, all the above-listed parameters were lowest on the upper slope gradient. In terms of soil depth, the sand fraction, TP, Av. P, TN, CEC, OM, extractable micronutrients like Fe, Mn, Cu, and Zn decreased with increasing soil depth, while the clay content, BD, pH, and exchangeable bases like Ca, Mg, Na, and K were increased with increasing soil depth. Regarding interaction effects, the highest TP, pH, OM, TN, AV.P, CEC, and extractable Mn, were observed on the lower slope gradient of surface (0–20 cm) soil depth. Generally, slope gradient and soil depth, and to some extent the interaction effect of slope gradient with soil depth, cause variations in physicochemical properties of the soil, exchangeable bases, and extractable micronutrients of the study area.

## 1. Introduction

Soil productivity is a critical factor in global food security, influencing agricultural output and sustainability. Across the world, soils face numerous challenges that limit their fertility and productivity, such as climate change, urbanization, and unsustainable agricultural practices. These challenges are compounded by socio-economic factors, including population growth and land-use pressures that vary by region [1,2]. In many developing countries, including those in Africa, the degradation of soil resources has reached alarming levels, threatening not only agricultural productivity but also the livelihoods of millions who depend on agriculture for their sustenance [3].

Agriculture is the backbone of Ethiopia’s economy, contributing 41% to the GDP, 84% to export earnings, and employing 80% of the population [4]. However, soil erosion, crop residue removal, and low inputs are among the leading causes of soil nutrient decline that threaten Ethiopian agriculture. According to [5], inappropriate land use and poor management have made soil degradation a global challenge for sustainable agricultural production. The primary determinant of sustainable soil productivity is the soil’s capacity to provide vital plant nutrients for plant development [6,7]. Massive land degradation has resulted from overgrazing and deforestation brought on by high population density. Due to anthropogenic and natural processes, land degradation has become a main policy concern in Ethiopia and is the reason for the high rate of nutrient depletion [8]. Land degradation is one of the challenges that has to be addressed immediately to boost agricultural production and ensure food security [9]. The various farms have various levels of soil fertility status due to differences in topography and nutrient status, which necessitate different management approaches. Topographies are among the soil-forming elements that affect how soil qualities are eroded by water [10]. Topography is one component of agricultural landscapes that requires different agronomic management and input levels [11]. Agricultural practices on steep slopes provide an ideal environment for soil erosion, which typically degrades the upper and middle soil and deposits it on the foot slopes [11,12].

Soil properties exhibit spatial variability over both small and large areas, influenced by intrinsic factors such as parent materials and climate, as well as extrinsic factors like soil management practices, inherent soil fertility, crop rotation, and the type of crops being grown [13,14]. Additionally, soil topography and depth play critical roles in shaping these properties, as different slope positions and soil depths can significantly affect moisture retention, nutrient availability, and erosion rates. Describing the spatial distribution of soil fertility across a field has become more manageable with the advent of technologies like Global Positioning Systems (GPS) and Geographic Information Systems (GIS). Utilizing GPS for soil sample collection is crucial for generating thematic soil fertility maps [15,16]. Likewise, GIS serves as a powerful tool for the effective access, retrieval, and manipulation of large datasets related to natural resources, which can be challenging to manage manually. It allows for the manipulation of both spatial and attribute data, making it easier to handle diverse data sources [7,17]. Numerous studies have employed geo-statistical analyses to explore the spatial variability of various soil properties [18,19]. Among the various geo-statistical techniques, Inverse Distance Weighting (IDW) is particularly favored for mapping soil fertility variation due to its superior prediction accuracy [20].

Soil fertility is a critical factor for successful agricultural production, particularly in irrigable areas such as the *Achewa* small-scale irrigation (SSI) scheme. Before implementing any SSI schemes, it is essential to assess the current status of soil fertility, where it is high, improved where it is low, and developed where it is lacking. This influences the effectiveness of agricultural practices.

While previous studies, such as those by [21,22], have examined the effects of land use change on soil nutrients in the *Itang* Special District, they primarily focused on the impact of land use changes on soil nutrients without evaluating the spatial and temporal variations of soil fertility. Therefore, ignoring slope position and soil depth may increase uncertainty in site-specific soil fertility management, especially in the study area.

In this context, mapping the spatial variability of soil fertility using geographic information systems has become essential to guide both current and future land management. Hence, the present study was planned to assess the soil fertility status and map the irrigable land of the *Achewa* Area, southwestern Ethiopia.

## 2. Materials and methods

### 2.1. Location

**Fig 1:**
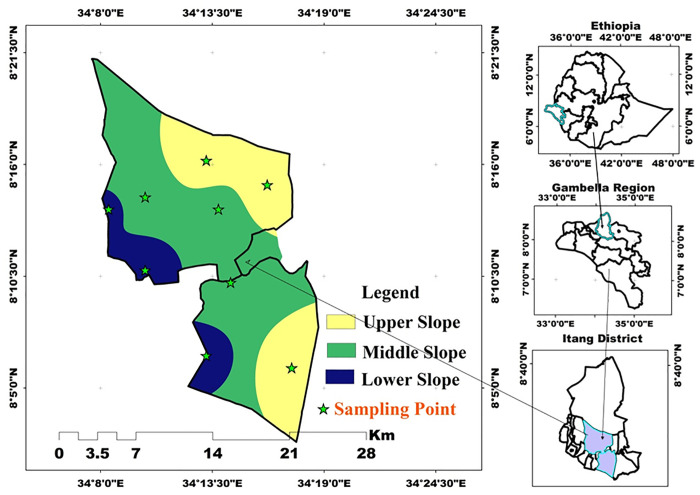
Map of the study area (Source: Developed by the researchers from ArcGIS 10.4) (2024).

The study was conducted at the *Achewa* irrigation site, located in *Itang* Special District of the Gambella People’s National Regional State (GPNRS) in southwestern Ethiopia (Fig 1). The study site is geographically positioned at approximately 8°12′36.96″N latitude and 34°14′32.92″E longitude, with a mean elevation of 424 meters above sea level (Masl). It is situated about 45 km west of Gambella town and approximately 770 km southwest of Addis Ababa (A.A). The landscape is predominantly flat to gently sloping, with slope gradients ranging from 0.2–0.5% in level areas to 5–10% in more undulating zones. These topographic conditions make the area suitable for small-scale irrigation development and soil water interaction studies.

### 2.2. Climate

**Fig 2:**
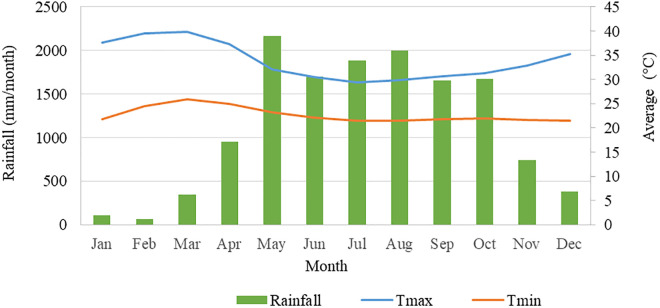
Monthly mean temperature and rainfall in the study Monthly mean temperature and rainfall in the study (Source: Ethiopian National Meteorological Agency of Gambella station).

The study location has a unimodal rainfall pattern, with April or June marking the onset of the wet season and extending through October, while February and March remain the driest months. Based on 2010–2020 meteorological data, the region receives an average annual rainfall of 1,024.9 mm, distributed irregularly across seasons, with mean temperatures ranging from 21.19°C to 39.84°C during the hot, dry season. Both rain-fed and irrigation-based farming systems are practiced, leaving smallholder farmers highly vulnerable to seasonal flooding during the wet season and prolonged dry spells during the dry months, which threaten crop yields and food security (Fig 2). The soils of the *Achewa* irrigation area are predominantly clay and clay loam in texture and are classified as Eutric Fluvisols (Loamic), Pellic Vertisols (Gilgaic, Hypereutric), and Haplic Vertisols (Gilgaic) [23].

### 2.3. Field survey and site selection

A reconnaissance study was conducted to get comprehensive technical details on the sample site and to acquire a clear visual representation of the topography and irrigation area history. Experts in the agricultural office in the district were participating in the field survey. The sample sites were categorized into three slope positions irrigation area based on a purposive selection method. Based on the proximity of the irrigation site to the river and the morphological properties of the soil were used for the selection of baseline assessment, and mapping soil fertility status was determined. The delineation of the study area was performed using the automatic delineation option of ArcGIS 10.8. According to [24], the slope classification system classified the area were classified into three categories for three different slope classifications: lower slope (0–2%), middle slope (2–5%), and upper slope (>5%). Both the Global Positioning System (GPS) and Clinometer were used to categorize the slope and pinpoint the precise slope positions of the soil sampled sites. Subsequently, the irrigable land was divided into three slope positions (lower, middle, and upper), two depths (0–20 and 20–40 cm), in triplicate, totaling eighteen composite soil samples collected.

Once a representative site was chosen, soil samples were obtained from three different slopes (Upper, Middle, and Lower) at two depths (0–20 cm and 20–40 cm), with three replications for each position. The samples were properly labeled, air-dried, and cleaned of any debris, like rocks and roots. The samples were subsequently crushed with a mortar and pestle and sieved through a 2 mm mesh for the majority of the physicochemical analyses, while organic carbon and total nitrogen samples were filtered through a 0.5 mm mesh. Ultimately, the 18 soil samples were sent to the soil laboratory at Arba Minch University for analysis, using established laboratory protocols.

### 2.4. Laboratory analysis

Soil particle size distribution was determined using the hydrometer method, subsequent to the procedure outlined by [25], and the soil texture class was identified based on the USDA textural triangle [26]. A digital pH meter was applied to determine soil pH at 1: 2.5 soil-to-water ratios. Analysis of organic carbon was conducted through [27], and soil organic matters were estimated by multiplying the percentage of the organic carbon by a factor of 1.724 [28]. A micro-Kjeldahl method to measure total nitrogen, considering digestion, distillation, and titration, was used as explained by [29]. Available phosphorus was determined using the Olsen method in combination with a spectrophotometer [30]. Exchangeable calcium (Ca²⁺) and magnesium (Mg²⁺) were analyzed using atomic absorption spectroscopy (AAS), while potassium (K⁺) and sodium (Na⁺) were measured with a flame photometer, as described by [31]. Cation exchange capacity (CEC) was assessed based on Chapman, (1965) method, following extraction with ammonium acetate. The following formula was used in measuring percent base saturation (PBS).


$$𝐏𝐁𝐒\;(\%)\;=(𝐂{𝐚}^{2+}+\;𝐌{𝐠}^{2+}+\;{𝐊}^{+}+\;𝐍{𝐚}^{+})/\;(𝐂𝐄𝐂)\;\times \;100.$$ (1)

Micronutrients such as iron (Fe), manganese (Mn), copper (Cu), and zinc (Zn) were extracted with diethylenetriaminepentaacetic acid (DTPA) and measured at their respective wavelengths using AAS, following the method of [16].

### 2.5. Statistical analysis

A Two-way analysis of variance (ANOVA) was conducted to determine whether there was significant difference in mean soil properties between depth and slope position in the *Achewa* Small-scale irrigation scheme. A two-way ANOVA test was used to examine the interaction effects of soil depth and slope gradients. A post-hoc analysis using the Tukey Honest Significant Difference (HSD) test was conducted to assess significant differences in mean soil properties among irrigable land soil depths and slope gradients at the 5% significance level. Furthermore, bivariate correlation analysis was carried out to illustrate the interrelationship among the physical and chemical properties of the key selected soils using SAS version 9.4 software [32]. Before performing the ANOVA, several assumptions were verified. The Shapiro-Wilk test confirmed the normality of the data, and Levene’s test evaluated the homogeneity of variances. Furthermore, Bartlett’s test of sphericity established significant correlations among the variables, supporting the validity of the analysis. The laboratory results, along with their corresponding geographic coordinates (latitude and longitude), were integrated into a GIS environment to examine the spatial variability of soil fertility throughout the study area.

### 2.6. Geostatistical soil fertility and mapping

The geographic coordinates (latitude and longitude) of each soil sampling point were recorded using a Garmin GPS device and imported into ArcGIS 10.5 to establish a georeferenced base map. Non-spatial soil data were linked to these coordinates and converted into spatial point layers representing 54 soil samples. To estimate values at unsampled locations, the Inverse Distance Weighted (IDW) interpolation method in the Spatial Analyst extension of ArcGIS was applied. IDW relies on the principle that the influence of a sampled point decreases with increasing distance, and thus, estimates are calculated as weighted means of neighboring values. Spatial maps illustrating the variability of soil physicochemical properties (pH, OM, TN, Av. P, CEC, and PBS) were generated using this technique. All geostatistical and mapping analyses were performed in ArcGIS 10.4. [33]. In the Inverse Distance Weighted (IDW) method, each measured point is assigned a weight inversely proportional to its distance from the prediction location, ensuring that closer points have greater influence on the estimated value. Unlike kriging, IDW does not rely on semivariograms or spatial structure modeling [34]. The accuracy of the interpolation was assessed through cross-validation using Mean Error (ME) and Root Mean Square Error (RMSE)

### 2.7. Ethics statement

Arba Minch University’s Research and Publication Directorate and Gambella University’s Research and Publication Directorate authorized the present study to collect soil samples and access the field site. Farmers agreed to collect the soil samples in the study area, as the survey has no harmful effects on humans.

### 2.8. Flow chart

**Fig 3:**
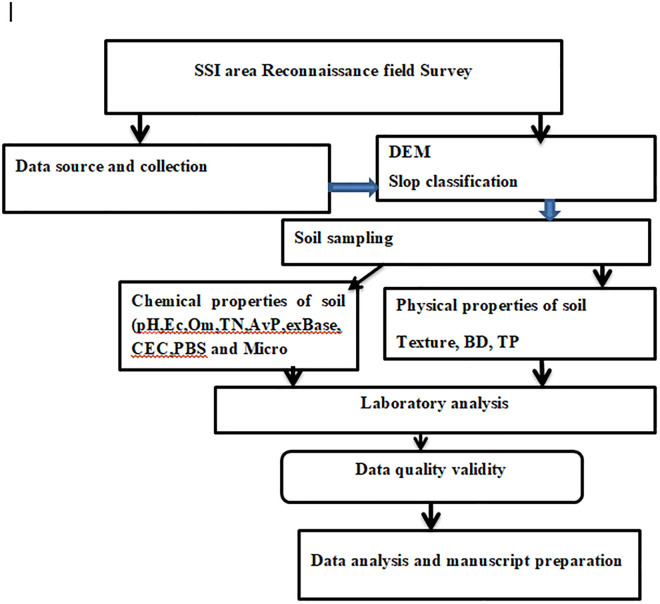
Research flow chart (Source: Developed by the researcher (2023).

The overall research process followed a structured sequence, as illustrated in (Fig 3). The flow chart outlines the key stages, beginning with problem identification and literature review, followed by site selection, sampling design, and data collection. Subsequent steps involved laboratory analysis, data processing, and statistical interpretation to evaluate the research objectives. Finally, results were synthesized, discussed, and compared with existing studies to draw meaningful conclusions and recommendations. Such flow charts are widely used in scientific studies to ensure methodological clarity and transparency [35]

## 3. Result

### 3.1. Assumptions of analysis of variance.

Shapiro-Wilk and Levene’s tests were employed to assess the normality of the data distribution and the homogeneity of variance, respectively [35]. The study found that all tested soil parameters were normally distributed, except for soil pH at a depth of, which had a W statistic of 0.790 and a p-value of 0.016, indicating a significant deviation from normality. Additionally, the homogeneity of variances for soil data collected from two soil depths and three slope locations in irrigable land met the assumptions for two-way ANOVA, as most significance values were greater than 0.05. However, the pH parameter did not satisfy the assumptions of homogeneity of equal variance, with a significance value of 0.025. This violation of equal variance was further evaluated using Welch’s robustness test. Overall, the results align with the findings of [35,36], who highlighted the importance of assessing homogeneity of variance when conducting ANOVA. In the present study, skewness values for all samples across soil depth and slope positions ranged between +1 and −1, indicating acceptable data distribution, except for EC and exchangeable Na in the slope position (Table 1).

**Table 1:** Test of normality and homogeneity of equal variance for soil physical and chemical properties in slope position and soil depth.

Soil parameters	Normality and homogeneity of variance of soil data					
	Shapiro-Wilk W test for normal data (n = 18) and Skewness					
	Soil Depth			Slope gradient		
	Statistic	Skewness	Sig.	Statistic	Skewness	Sig.
Sand (%)	.839	0.11	.057	.882	0.705	.167
Silt (%)	.901	−0.34	.260	.974	0.173	.930
Clay (%)	.843	0.668	.062	.887	−0.881	.185
BD g/cm^3^	.879	−0.54	.154	.957	−0.390	.768
TP (%)	.891	0.49	.205	.961	0.373	.811
pH (H_2_0)	.790	0.95	.016*	.952	−0.552	.715
EC	.894	−.040	.218	.808	−1.778	.025
OM (%)	.962	0.059	.818	.893	−0.233	.217
OC (%)	.962	0.075	.821	.894	−0.225	.217
TN (%)	.935	0.45	.531	.851	−0.574	.076
AvP (ppm)	.921	0.22	.399	.961	−0.068	.814
Ca^2 + ^ (cmol(+)kg^-1^)	.914	0.559	.341	.866	0.991	.111
Mg^2+^ (cmol(+)kg^-1^)	.927	−0.89	.457	.895	−0.267	.225
Na^+^ (cmol(+)kg^-1^)	.947	0.033	.662	.882	−1.039	.166
K^+^(cmol(+)kg^-1^)	.966	0.327	.855	.961	0.522	.804
CEC (cmol(+)kg^-1^)	.906	−0.005	.287	.955	0.100	.742
PBS (%)	.946	0.636	.647	.990	0.130	.996
Fe	.851	0.866	.076	.954	0.549	.737
Mn	.849	0.777	.074	.918	0.164	.378
Cu	.968	0.65	.881	.973	0.413	.921
Zn	.954	0.72	.739	.914	0.199	.343

*Significant at p < 0.05, not statistically significant, n number of samples

#### 3.1.1. Effects of slope gradients and soil depths on selected physicochemical properties of soil.

**Soil particle size distribution** The analysis of soil particle size distribution revealed significant variations influenced by slope gradients and soil depths. At the lower slope, both surface and subsurface soil layers exhibited the highest clay content at 58.17%, while the lowest sand content was recorded at 17.00%. In contrast, the middle slope displayed the highest mean sand percentage at 34.76%, and the upper slope had the lowest clay percentage at 36.50%. Overall, clay content increased from the upper to the lower slopes, while sand content decreased, indicating a trend consistent with findings by [37].

Regarding soil depth, the surface layer (0–20 cm) showed a higher sand fraction of 30.05% and lower clay content at 42.83%. Conversely, the subsurface layer (20–40 cm) exhibited a higher clay content of 46.56% and lower sand content of 25.78%. This trend demonstrates that clay content increases with depth, aligning with observations by [38]. Additionally, bulk density (BD) was lowest at 1.29 g/cm³ in the lower slope and highest at 1.37 g/cm³ in the upper slope, highlighting the effect of slope on soil compaction.

Clay, silt, and sand fractions were significantly affected by the interaction of slope and soil depth (Table 3). The highest clay content (61.00%) was observed in the subsurface of the lower slope, whereas the lowest (32.34%) occurred in the surface of the upper slope. In contrast, the greatest sand fraction (36.17%) was recorded in the surface of the middle slope, while the lowest (15.33%) was found in the subsurface of the lower slope. Similarly, silt content was highest in the surface of the upper slope (32.33%) and was lowest in the subsurface of the lower slope (23.05%).

**Table 3:** Two-way ANOVA results for the interaction effects of slope position and soil depth on soil chemical properties in the Achewa irrigated area.

Slope with soil depth	Soil Physical Properties					
	Texture				TP (%)	BD (g cm^-3^)
		Clay%	Silt%		Sand%	Classes
Upper slope * D_1_	32.34^**d**^	32.33^**a**^	35.33^**a**^	Clay Loam	49.31^**c**^	1.34^**b**^
Upper slope * D_2_	40.67^**c**^	30.67^**a**^	28.66^**b**^	Clay	47.17^**d**^	1.40^**a**^
Middle slope * D_1_	40.83^**c**^	23.67^**c**^	36.17^**a**^	Clay	50.94^**ab**^	1.30 cd
Middle slope * D_2_	38.00^**c**^	28.67^**ab**^	33.33^**ab**^	Clay Loam	47.42^**d**^	1.39^**a**^
Lower slope * D1	55.33^b^	26.00^bc^	18.67^**c**^	Clay	51.82^**a**^	1.27^**d**^
Lower slope * D_2_	61.00^**a**^	23.05^**c**^	15.33^**c**^	Clay	50.81^**b**^	1.32^**c**^
CV (%)	5.37	9.10	9.27		1.33	1.30
P-value	**	*	Ns		*	*

Means values sharing the same letter in the same (column or row) do not differ statistically at the specified levels of significance.D1 = depth one (0–20), D2 = depth two (20–40 cm). From this ** = highly significant at P < 0.01, *** = Very highly significant at P < 0.001, Ns = Non-significant at P > 0.05, CV = Coefficient of variation, LSD = Least significant difference and P = Probability

**Soil Bulk Density (BD)** Bulk density was significantly influenced by slope position and soil depth (p < 0.001). The lowest BD (1.29 g cm ⁻ ³) was recorded on the lower slope at 0–20 cm depth, while the highest (1.37 g cm ⁻ ³) occurred on the upper slope at 20–40 cm depth. According to [39], these values are categorized as low on lower slopes and medium on middle and upper slopes. The increase in BD with slope and depth is linked to lower organic matter content and compaction, consistent with earlier findings [37,40].

**Total Porosity (TP)** TP was highly significantly affected by slope and soil depth (p < 0.001). The highest TP (51.32%) was observed at the lower slope, while the lowest (48.24%) was recorded at the upper slope. Similarly, surface soils had higher TP (50.69%) compared to subsurface soils (48.47%). These results agree with the inverse relationship between BD and TP reported by [38], highlighting the role of organic matter and aggregation in enhancing porosity.

#### 3.1.2. Effects of slope and soil depth on soil chemical properties.

**Fig 4:**
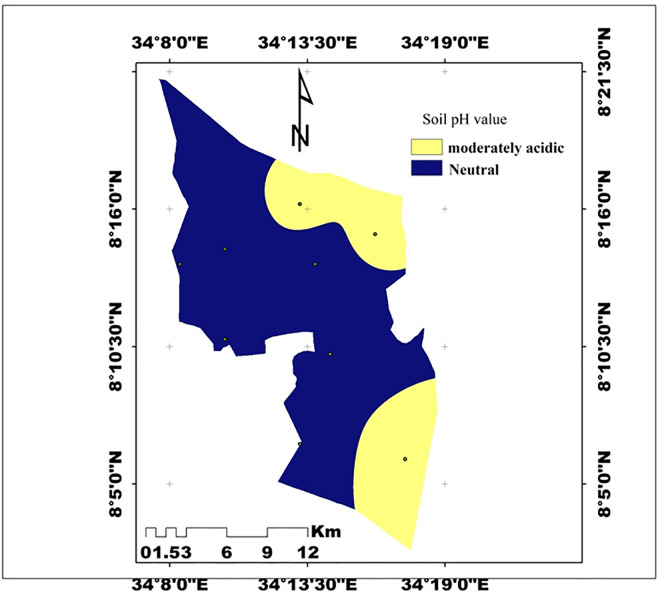
Spatial distribution of soil pH in the study area (Source: Developed by the researchers from ArcGIS 10.4) (2024).

**Soil pH** Soil pH was significantly influenced by slope position and depth (p < 0.05). The mean pH increased downslope, ranging from 6.23 at the upper slope to 7.28 at the lower slope, and was slightly higher in subsurface soils (6.98) compared to surface soils (6.80). According to [41], soils in this range are classified as moderately acidic to neutral (Fig 4). The rise in pH along the downslope gradient can be explained by the mobilization of exchangeable base cations from upper slope positions and their subsequent accumulation in lower landscape positions, which promotes alkalinity. These findings are consistent with the results of [42], who reported relatively higher pH values at lower slope positions.

**Electrical Conductivity (EC)** EC was not significantly affected by slope position, soil depth, or their interaction (p > 0.05). Numerically, the highest EC (1.64 dS/m) was recorded at the lower slope and the lowest (1.41 dS/m) at the upper slope. According to [43], all EC values fall within the non-saline range (<2 dS/m), suggesting no salinity constraints for crop production. The slightly higher EC at lower slopes may be linked to soluble salt and nutrient deposition, as also reported by [44].

**Organic Matter (OM)** OM content was significantly affected by both slope and depth (p < 0.01). The highest mean OM (3.52%) was found at the lower slope, while the lowest (2.60%) was observed at the upper slope. Surface soils (3.45%) contained more OM than subsurface soils (2.69%). According to [39], these values fall within medium to high fertility ranges. The decline in OM with slope and depth reflects erosion losses and reduced organic inputs, in agreement with [45], who reported similar findings.

**Fig 5:**
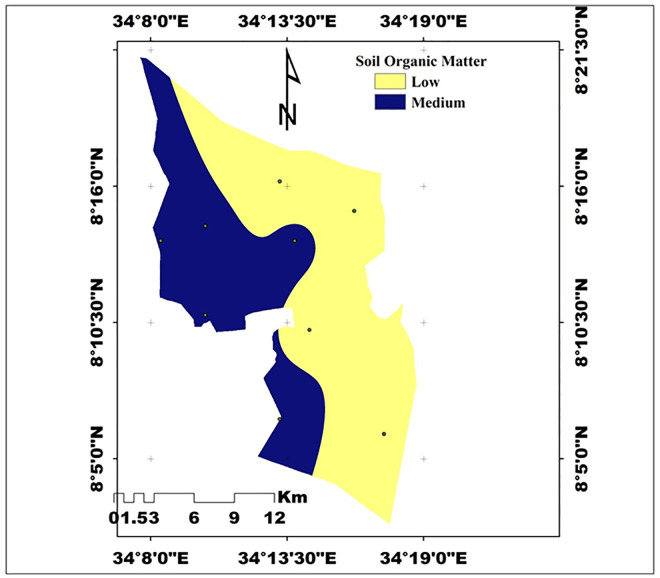
Spatial distribution of OM in the study (Source: Developed by the researchers from ArcGIS 10.4) (2024).

**Total Nitrogen (TN)** TN varied significantly across slope and depth (p < 0.01). The lower slope recorded the highest TN (0.21%), followed by the middle (0.19%) and the upper slope (0.16%). Surface soils (0.20%) contained more TN than subsurface soils (0.17%). According to [41], TN is rated medium at the lower and middle slopes and low at the upper slope (Fig 5). The variability in TN across the study sites reflects its strong association with soil organic matter (OM), which functions as the dominant source and sink of nitrogen in soils. This observation corroborates the work of [46], who reported similar results.

Available Phosphorus (Av.P): Av. P was significantly influenced by slope position and depth (p < 0.05). The highest Av. P (26.46 mg/kg) was recorded in the lower slope surface soils, while the lowest (19.44 mg/kg) occurred in the upper slope subsurface soils. According to Olsen’s classification [47], these values are rated high. The higher content of available phosphorus in the farm might be due to the soil parent material of the soil and the climatic aridity of the study area may favour the concentration of higher available phosphorus. Similar findings were reported in the Gambella region [48,49], which reported higher available P values in the western Ethiopia Gambella region soil.

Exchangeable Bases: Exchangeable Ca, Mg, Na, and K were significantly influenced by slope (p < 0.05; Table 5). Ca (8.55 mg/l), Na (0.69 mg/l), and K (0.43 mg/l) peaked at the lower slope, while Mg (6.14 mg/l) was highest at the middle slope. The lowest values of Ca (6.23 mg/l), Mg (4.66 mg/l), and K (0.28 mg/l) occurred at the upper slope, whereas Na was lowest at the middle slope (0.41 mg/l). The increase of bases downslope is linked to clay and CEC enrichment, consistent with [42,50].

**Table 5:** Results of the two-way ANOVA showing the main effects of slope position and soil depth on soil chemical properties in the Achewa irrigated area.

Slope gradient* Soil depth	pH (H_2_O)	EC (ds/m)	OM	TN	Av.p (ppm)
			(%)	(%)	
Upper slope * D_1_	5.95^**c**^	1.29^**b**^	1.56^**c**^	0.20^**b**^	21.28^**c**^
Upper slope * D_2_	6.52^**b**^	1.50^**ab**^	1.47^**c**^	0.12^**c**^	19.44^**d**^
Middle slope * D_1_	7.14^a^	1.69^**a**^	3.56^**b**^	0.20^**b**^	24.25^**b**^
Middle slope * D_2_	7.15^**a**^	1.48^**ab**^	1.52^**c**^	0.15^**c**^	20.43 cd
Lower slope * D_1_	7.30^**a**^	1.80^**a**^	4.13^**a**^	0.26^**a**^	26.46^**a**^
Lower slope * D_2_	7.25^**a**^	1.49^**ab**^	2.91^**c**^	0.16^**c**^	25.55^**a**^
CV (%)	2.33	13.18	8.47	10.30	3.82
P-value	*	Ns	*	*	*

Means with the same letter are not significantly different from each other.D1 = depth one (0–20), D2 = depth two (20–40 cm), Ns = Non-significant at P > 0.05, CV = Coefficient of Variation, P = Probability, and LSD = Least significant difference

Cation Exchange Capacity (CEC): CEC was significantly affected by slope, depth, and their interaction (p < 0.01). The maximum CEC (32.29 cmol(+)/kg) was observed in surface soils of the lower slope, while the minimum (19.85 cmol(+)/kg) was found in subsurface soils of the upper slope. According to [41], the soils are rated high in lower and middle slopes and optimum at the upper slope. Higher OM and clay content explain the higher CEC at lower slopes, consistent with [49].

Percentage Base Saturation (PBS): PBS was significantly influenced by soil depth and slope with depth interaction (p < 0.01), but not by slope alone (p > 0.05). The highest PBS (75.05%) was recorded in subsurface soils of the lower slope, while the lowest (35.32%) occurred in surface soils of the upper slope. These results reflect cation leaching and deposition processes. This result is in agreement with [44], who reported similar findings in the Gumara Watershed of the Lake Tana Basin, Northwestern Ethiopia.

Extractable micronutrients: Micronutrients (Fe, Mn, Cu, Zn) were significantly affected by slope and depth (p < 0.05). Surface soils contained higher concentrations Fe (17.43 mg/kg), Mn (10.96 mg/kg), Cu (3.03 mg/kg), Zn (2.15 mg/kg)] compared to subsurface soils [Fe (9.98 mg/kg), Mn (7.00 mg/kg), Cu (2.03 mg/kg), Zn (1.51 mg/kg)]. Among slope positions, Fe (15.78 mg/kg), Mn (11.08 mg/kg), and Cu (2.82 mg/kg) were highest in the lower slope, while Zn (1.97 mg/kg) was slightly higher at the middle slope. According to critical levels set by [41], Fe and Mn were sufficient, while Cu and Zn were near the deficiency threshold, highlighting the need for micronutrient management. This research is consistent with the findings of [3], who reported similar patterns in the Ayiba Watershed of the Northern Highlands, Ethiopia.

### 3.2. Discussion

#### 3.2.1. Effects of slope and soil depth on soil physical properties.

**Particle size distribution**: The variation in soil particle size distribution observed in the *Achewa* irrigated area reflects the combined influence of slope positions and soil depth. The dominance of clay in the lower slope positions and the prevalence of clay loam in the middle and upper slopes suggest a strong role of topography in governing soil redistribution. This agrees with the general understanding that finer particles, such as clay and silt, are easily detached and transported downslope by water erosion, leading to their accumulation in lower-lying areas, while coarser fractions, including sand, tend to remain in upper positions [12,51].

Considering the main effects of slope position, the lower slope had the lowest mean sand content (17.00%) and the highest mean clay content (58.17%). In contrast, the middle slope exhibited the highest sand fraction (34.76%), while the upper slope had the lowest clay content (36.50%) (Table 2). Overall, clay content increased progressively from the upper to the lower slopes, whereas both sand and silt contents decreased along the same gradient. This trend can be attributed to topography-driven soil redistribution processes, whereby finer particles such as clay are detached from the upper slope through erosion and subsequently deposited in the lower slope positions. In contrast, coarser fractions, particularly sand, tend to remain in the higher slope positions due to their lower susceptibility to transport.

**Table 2:** Two-way ANOVA results for the interaction effects of slope position and soil depth on soil physical properties in the Achewa irrigated area.

Slope	Particle size distribution Texture				TP (%)	BD (g cm^-3^)
	Clay%	Silt%	Sand%	Textural Classes		
Upper slope	36.50^**c**^	31.50^**a**^	32.00^**a**^	Clay Loam	48.24^**c**^	1.37^a^
Middle slope	39.41^**b**^	25.83^**b**^	34.76^**a**^	Clay Loam	49.18^**b**^	1.34^**b**^
Lower slope	58.17^**a**^	24.83^**b**^	17.00^**b**^	Clay	51.32^**a**^	1.29^**c**^
LSD (0.05%)	2.020	3.137	3.258		0.831	0.022
CV (%)	5.37	9.10	9.27		1.33	1.30
P-value	***	**	***		***	***
Soil depth in centimeters (cm)						
0-20 (Surface)	42.83^**b**^	27.12^**a**^	30.05^**a**^	Clay	50.69^**a**^	1.30^**b**^
20-40 (Sub_ surface)	46.56^**a**^	27.66^**a**^	25.78^**b**^	Clay	48.47^**b**^	1.36^**a**^
LSD (0.05%)	2.465	2.562	2.660		0.678	0.018
P-value	**	Ns	***		***	***

Means values sharing the same letter in the same (column or row) do not differ statistically at the specified levels of significance.D1 = depth one (0–20), D2 = depth two (20–40 cm). From this ** = highly significant at P < 0.01, *** = Very highly significant at P < 0.001, Ns = Non-significant at P > 0.05, CV = Coefficient of variation, LSD = Least significant difference and P = Probability

The significant effect of slope and soil depth on clay and sand fractions also indicates that vertical and lateral soil development processes are actively shaping soil texture. At lower slopes, the higher clay content enhances water-holding capacity and nutrient retention, which may benefit crop production under irrigation. However, such soils are also prone to poor drainage and compaction, which can restrict root penetration and reduce aeration [52]. Conversely, the relatively lighter texture at the middle and upper slopes provides better drainage and aeration, but the reduced clay fraction may limit nutrient and water availability, potentially increasing the risk of crop moisture stress, particularly under rainfed conditions. The overall dominance of clay and clay loam textures in the study area indicates that soil formation is strongly influenced by the nature of the parent material. Similar findings have been reported in the Abobo Agricultural Research Site [37] and other irrigated lowland areas of Ethiopia [53,54], where clay-rich soils were associated with alluvial deposits and topographic redistribution. This suggests that the *Achewa* soils share common geomorphological and pedogenic processes with other lowland systems in the region.

#### 3.2.3. Soil bulk density and total porosity.

**Soil bulk density (BD)**: The BD value in the *Achewa* irrigated area was significantly affected by slope position (p < 0.001), soil depth (p < 0.001), and their interaction (p < 0.05) (Table 2). BD ranged from 1.29 g cm ⁻ ³ at the lower slope to 1.37 g cm ⁻ ³ at the upper slope. The soils of the higher slope position may have the highest bulk density due to low organic matter content and compaction from repeated cultivation. This result is consistent with the findings of multiple authors, including [44,48,55], who reported that variations in the amounts of clay fraction and organic matter were the cause of low and high bulk density values observed in the lower and upper slope positions, respectively. Similarly, [42] observed that the BD on upper slopes was highest at 1.41 g cm ⁻ ³ compared to 1.32 g cm ⁻ ³ on lower slopes. In contrast, the lower slope accumulates eroded sediments and organic matter, resulting in improved aggregation and reduced BD. Surface soils (0–20 cm) had lower BD (1.27–1.32 g cm ⁻ ³) than subsurface layers (20–40 cm, up to 1.40 g cm ⁻ ³), reflecting higher organic matter inputs and biological activity near the surface, while overburden pressure and lower OM in deeper horizons contribute to compaction.

The interaction between slope position and soil depth further accentuated these trends, with the maximum bulk density (1.40 g cm ⁻ ³) occurring in the subsurface soils (20–40 cm) (Table 3) of the upper slope, whereas the minimum value (1.27 g cm ⁻ ³) was recorded in the surface soils (0–20 cm) of the lower slope. This indicates that upper slopes are more vulnerable to erosion-induced compaction, whereas lower slopes benefit from deposition. Although some studies report higher BD at lower slopes, others align with our findings, underscoring the site-specific nature of BD variation. According to [39], *Achewa* soils are classified as low to medium BD, suggesting generally favorable porosity and root penetration. However, elevated BD in upper slope subsurface soils may limit water infiltration and root growth if unmanaged. These results highlight the need for slope-sensitive management practices, including erosion control and organic matter amendments, to sustain soil quality and irrigation productivity in the region.

The average total porosity (TP) values of soils at the upper, middle, and lower slope positions were 48.24%, 49.18%, and 51.32%, respectively (Table 2). This shows that TP increased downslope, with the highest porosity recorded in the lower slope and the lowest in the upper slope. Soil depth also significantly affected TP (p < 0.001), where surface soils (0–20 cm) had higher TP (50.69%) than subsurface soils (20–40 cm), which recorded 48.47%. The interaction between slope position and soil depth was also significant (p < 0.05). The highest TP (51.82%) was observed in the surface soils of the lower slope, while the lowest TP (47.17%) was recorded in the subsurface soils of the upper slope. These findings indicate that total porosity is strongly influenced by both topographic position and soil depth, with higher porosity at lower slopes likely resulting from deposition of finer particles and greater organic matter accumulation. Overall, TP values across the study area remained above 40%, classifying them as very high according to [43].

### 3.3. Soil chemical properties

#### 3.3.1. Soil reaction (pH).

Soil pH was significantly affected by slope position (p < 0.001), soil depth, and their interaction (p < 0.05) (Table 4). Across slope gradients, the mean values were 7.28, 7.15, and 6.23 for the lower, middle, and upper slopes, respectively. Soil pH was significantly lower at the upper slope positions compared to the middle and lower slopes. This can be explained by the greater loss of basic cations through leaching and surface runoff on upper slopes, which leads to soil acidification. In contrast, lower slope positions tend to accumulate these cations, resulting in relatively higher soil pH. Similar findings were reported by [56] in Mawula Watershed, Loma District, Southern Ethiopia, [57] in the Guder watershed, [58] in the Zikre watershed, and [59] in the Rift Valley, all of whom reported that soils on steeper slopes tend to be more acidic as a consequence of enhanced leaching and erosion. Similarly, [60]in the Ele watershed, and [55] in the Karnuary watershed, also confirmed that soil pH increases downslope due to the accumulation of base cations in lower slope positions. According to the classification proposed by [61] (Fig 4), soils of the study area are rated as moderately acidic on the upper slope and neutral on both the middle and lower slopes.

**Table 4:** Two-way ANOVA results showing the interaction effects of slope position and soil depth on chemical properties of soil in the Achewa irrigated area.

Sampling site	pH (H_2_O)	EC (ds/m)	OM (%)	TN (%)	Av. P (ppm)
Upper slope	6.23^**b**^	1.39^**a**^	2.60^**c**^	0.16^**b**^	20.36^**c**^
Middle slope	7.15^**a**^	1.59^**a**^	3.10^**b**^	0.17^**b**^	22.34^**b**^
Lower slope	7.28^**a**^	1.64^**a**^	3.52^**a**^	0.21^**a**^	26.00^**a**^
LSD (0.05%)	0.2021	0.256	0.328	0.023	1.102
P-value	***	Ns	***	**	***
Soil depth in centimeters (cm)					
0-20	6.80^**b**^	1.59^**a**^	3.45^**a**^	0.22^**a**^	24.00^**a**^
20-40	6.98^**a**^	1.49^**a**^	2.69^**b**^	0.14^**b**^	21.80^**b**^
LSD (0.05%)	0.165	0.209	0.267	0.019	0.900
CV (%)	2.33	13.18	8.47	10.30	3.82
P-value	*	Ns	***	***	***

Means values sharing the same letter in the same (column or row) do not differ statistically at the specified levels of significance. From this ** = highly significant at P < 0.01, *** = Very highly significant at P < 0.001, Ns = Non-significant at P > 0.05, CV = Coefficient of variation, LSD = least significant difference, and P = Probability.

Soil depth also had a significant effect on soil pH (p < 0.05). The subsurface layer (20–40 cm) had a higher average pH (6.98) compared to the surface layer (0–20 cm), which recorded 6.80 (Table 4). This trend can be attributed to the higher concentration of exchangeable bases at depth, as basic cations tend to leach downward from surface horizons, resulting in lower surface pH. Comparable observations were made by [7,62–64], all of whom reported an increasing trend of pH with soil depth. Similarly, [65] explained that leaching of bases reduces surface soil pH relative to subsurface horizons. These results align with [66] along a toposequence in southern Ethiopia also confirmed a general increase in soil reaction with depth, who confirmed that soil reaction increases with depth. Following [61] classification, soils in both the surface (0–20 cm) and subsurface (20–40 cm) layers of the study area are rated as neutral.

The interaction between slope position and soil depth had a significant effect on soil pH values in the study area (P ≤ 0.05) (Table 5). The highest mean pH (7.30) was recorded in the surface soil (0–20 cm) of the lower slope, while the lowest mean pH (5.93) was observed in the sub-surface soil of the upper slope (Table 5). This variation can be attributed to erosion processes, where fine particles and basic cations are removed from upper slope positions and deposited downslope. Similar findings were reported by [60] in the Ele watershed and [55] in the Karnuary watershed, where erosion and runoff were shown to enhance the leaching of basic cations, thereby lowering soil pH and increasing acidity. This phenomenon is largely explained by the depletion of exchangeable bases caused by the continual removal of surface soil through erosion and runoff. According to [61], soils at middle and lower slope positions are generally classified as neutral, whereas upper slope soils tend to remain moderately acidic (Fig 4), consistent with the greater susceptibility of steeper slopes to leaching and acidification.

#### 3.3.2. Electrical Conductivity (EC).

The electrical conductivity (EC) values of soils in the *Achewa* SSI sites were not significantly affected by slope position, soil depth, or their interaction (p > 0.05). However, numerical differences provide important insights into the dynamics of soluble salts across the landscape. The relatively higher EC observed on the lower slope (1.64 dS/m) compared to the upper slope (1.39 dS/m) can be attributed to the downslope movement and accumulation of dissolved salts through runoff and leaching processes. This pattern is consistent with the general understanding that lower slope positions often act as depositional zones for both sediments and soluble ions [58,67]. Similarly, the decreasing trend of EC with soil depth, where surface soils recorded slightly higher values (1.59 dS/m) than subsoil layers (1.49 dS/m), though statistically insignificant, reflects the influence of evapotranspiration and surface salt accumulation. Shallow soil layers are more exposed to evaporation, which tends to concentrate soluble salts at the surface, whereas deeper layers are less affected [57,60]. This result suggests that while the overall salinity level of the soils in the study area is within a safe range for crop production, localized accumulation of salts at the surface and in lower slope positions warrants attention under continuous irrigation.

#### 3.3.3. Soil organic matter.

The analysis of organic matter (OM) reveals significant variations based on slope position and soil depth. In general, the OM values in the study area were higher across all slope positions, with the highest recorded at 3.52% in the lower slope position and the lowest at 2.60% in the upper slope position (Fig 5). The cause for the higher mean value of OM that was detected from the lower slope of the study area might be linked to the removal of organic matter from the upper slope to the lower slope. This is consistent with the findings of [59,66,68], who indicated that the quantity of soil organic matter (SOM) was higher at the middle slope and lower slopes compared to the upper slope. Moreover, the decline in soil organic matter (OM) content in the upper slope of the study area is evident, with OM values generally lower compared to other slope positions. This reduction can be linked to intensive cultivation practices and erosion, both of which significantly deplete organic matter and compromise soil health.

Similarly, SOM was greater in the surface layer (0–20 cm, 3.45%) compared to the subsurface layer (20–40 cm, 2.69%), reflecting an increase of 28.3%. This pattern is likely due to greater accumulation of organic residues and enhanced microbial activity in surface soils and depositional lower slopes, whereas upper slopes and deeper layers experience reduced organic input and higher losses from erosion and leaching. Similar trends have been reported in Ethiopian landscapes, where SOM is concentrated in surface soils and lower slope positions, while upper slopes and subsurface horizons show lower organic matter due to diminished biomass input and increased oxidation [55,69,70]. According to the classification by [71] indicates that SOM in the study area is medium on lower and middle slopes but low on upper slopes, highlighting the vulnerability of steep slope soils to fertility decline (Table 4).

#### 3.3.4. Total nitrogen.

Total nitrogen (TN) was significantly affected (P < 0.05) by the interaction of slope position and soil depth. The highest TN (0.26%) was observed in the surface soil (0–20 cm) of the lower slope, while the lowest TN (0.12%) occurred in the subsurface soil (20–40 cm) of the upper slope. Intermediate values were recorded in the middle slope, with 0.20% in the surface layer and 0.15% in the subsurface (Table 4). These differences reflect the combined effects of nutrient accumulation, microbial activity, and organic matter inputs in depositional lower slopes and surface soils, whereas upper slopes and deeper layers experience reductions in TN due to erosion, leaching, and limited biomass input. The soil erosion brought on by heavy rainfall, inadequate input application, crop residue removal, continuous cultivation, and the steepness of the slope in irrigated areas might be the cause of the upper slopes’ markedly lower TN content when compared to the upper, middle, and lower slope positions. This result is in line with the findings [56,70] who indicated that the loss of nitrogen was caused by the total clearance of crop residue from the field and continued cultivation, which aggravates the quick rate of mineralization in Mawula Watershed, Loma District, Southern Ethiopia, and in Debre-Mewi Watershed, northwestern Ethiopia, respectively.

**Fig 6:**
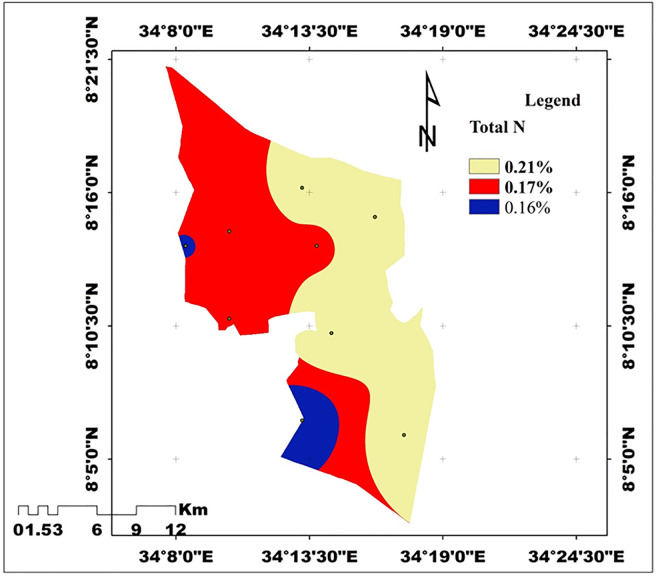
Spatial distribution of TN in the study (Source: Developed by the researchers from ArcGIS 10.4) (2024).

Considering the interaction of slope position and soil depth (Table 6), the highest total nitrogen (TN) (0.26%) occurred in the surface soil (0–20 cm) of the lower slope, followed by the surface soil of the upper slope (0.20%). The lowest values were recorded in the subsurface soil (20–40 cm) of the upper (0.12%) and lower slopes (0.16%). This pattern reflects greater TN accumulation in surface soils of depositional lower slopes, where organic matter input, microbial activity, and moisture availability enhance nitrogen retention, whereas upper slopes and deeper layers experience losses through erosion, leaching, and limited biomass input. According to [41], TN in the upper-slope subsurface soil is rated low, while values in the lower and middle slopes are within the optimum range (Fig 6).

**Table 6:** Results of the two-way ANOVA showing the main effects of slope position and soil depth on soil chemical properties in the Achewa irrigated area.

Sampling site	Exchangeable bases in mg/l				CEC (Cmol (+)/kg)	PBS (%)
	Ca^2+^	Mg^2+^	Na^+^	K^+^		
Upper slope	6.23^**c**^	4.66^**b**^	0.60^**a**^	0.28^**b**^	22.26^**c**^	55.13^**a**^
Middle slope	7.21^**b**^	6.14^**a**^	0.41^**b**^	0.36^**ab**^	25.90^**b**^	55.22^**a**^
Lower slope	8.55^**a**^	6.05^**a**^	0.69^**a**^	0.43^**a**^	28.96^**a**^	56.44^**a**^
LSD (0.05%)	0.751	0.666	0.129	0.061	1.037	4.382
P-value	***	***	**	***	***	Ns
Soil depth in centimeters (cm)						
0_20	5.70^**b**^	4.37^**b**^	0.48^**b**^	0.32^**b**^	28.08^**a**^	38.69^**b**^
20_40	8.96^**a**^	6.89^**a**^	0.65^**a**^	0.40^**a**^	23.33^**b**^	72.50^**a**^
LSD (0.05%)	0.613	0.544	0.108	0.050	0.846	3.577
CV (%)	8.15	9.42	18.61	13.54	3.20	6.26
P-value	***	***	**	*	***	***

Means values sharing the same letter in the same (column or row) do not differ statistically at the specified levels of significance. From this ** = highly significant at P < 0.01, *** = Very highly significant at P < 0.001, Ns = Non-significant at P > 0.05, CV = Coefficient of variation, LSD = Least significant difference and P = Probability, CEC = cation exchange capacity and PBS = Percent Base Saturation

#### 3.3.4. Available phosphorus (Av P).

The analysis of available phosphorus (Av. P) reveals significant variations based on slope position and soil depth. In general, the available P values in the study area were higher across all slope positions, with the highest recorded at 26.00 mg/kg in the lower slope position and the lowest at 20.36 mg/kg in the upper slope position. Remarkably, the mean Av. P content increased from the upper slope toward the lower slope of the watershed. This trend may be attributed to the removal of nutrient-rich topsoil, along with relatively high levels of organic matter, soil pH, and clay content found at lower slope positions. Such factors contribute to enhanced phosphorus availability, supporting plant growth and soil health. Furthermore, when considering soil depth, the surface soil (0–20 cm) recorded the highest Av. P value of 24.00 mg/kg, surpassing the subsurface soil (20–40 cm), which had an Av. P of 21.80 mg/kg. These findings illustrate that both slope position and soil depth significantly influence phosphorus availability, with lower slopes and shallower depths providing higher levels of this essential nutrient (Table 4).

The soil Av. P trend was as follows of soil organic matter and continued as follows: lower slope > middle > Upper slope; in terms of soil depth, surface soil > sub-surface soil positions (Table 4). The higher content of available phosphorus in the farm might be due to the soil parent material of the soil and the climatic aridity of the study area may favor the concentration of higher available phosphorus. Similar findings were reported in the Gambella region [48,72], which reported higher available P values in the western Ethiopian Gambella region soil. It was also reported that P availability in the soils might have been favored by the warm climatic conditions of the study area, along with the preferred pH range [73].

**Fig 7:**
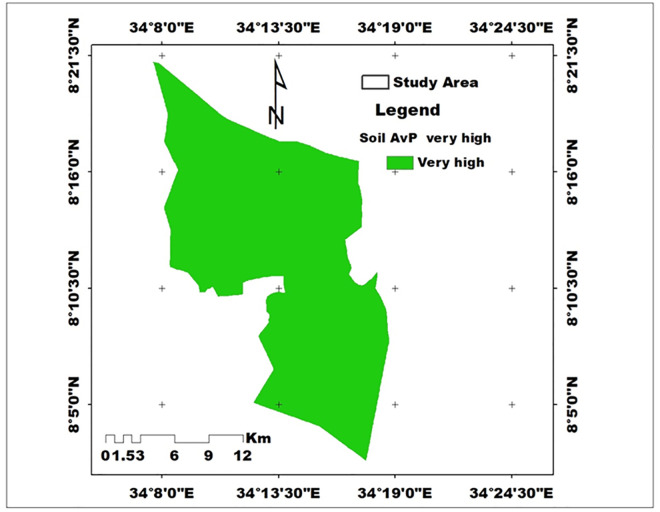
Spatial distribution of available phosphorus in the study area (Source: Developed by the researchers from ArcGIS 10.4) (2024).

Based on the rating suggested by [30], the available P contents of soil in the study area were found to be high (Fig 7), indicating that available P would not be a limiting nutrient for agricultural production in the area.

Considering the interaction between slope position and soil depth indicated that the highest available phosphorus (Av.P) content (26.46 mg/kg) was recorded in the surface soil (0–20 cm) of the lower slope, followed by the subsurface soil (20–40 cm) of the lower slope (25.55 mg/kg) (Table 5). In the middle slope, Av. P was 24.25 mg/kg in the surface layer and 20.43 mg/kg in the subsurface, whereas in the upper slope, the surface soil contained 21.28 mg/kg and the subsurface 19.44 mg/kg. These results demonstrate that depositional lower slopes and surface soils accumulate higher phosphorus due to nutrient deposition and organic matter inputs, whereas upper slopes and subsurface layers are more prone to nutrient loss through leaching and runoff, resulting in comparatively lower Av. P levels.

#### 3.3.6. Exchangeable bases.

The exchangeable cations (Ca^2+^, Mg^2+^, Na^+^, and K^+^) were significantly (P ≤ 0.05) affected by slope position, soil depth, and the interaction between slope and soil depth (Tables 6–7). In general, mean concentrations of all cations were significantly higher in the lower slope compared with the middle and upper slopes (Tables 6 and 7). The highest mean values of Ca (8.55 mg/l), Na (0.69 mg/l), and K (0.43 mg/l) were observed at the lower slope, while Mg^2+^ did not show a significant difference; it was highest on the middle slope (6.14 mg/l) and lowest upper slope (4.66 mg/l). In contrast, the lowest values of Ca^2+^ (6.23 mg/l), Mg^2+^ (4.66 mg/l), and K (0.28 mg/l) occurred on the upper slope, and the lowest Na (0.41 mg/l^2+^) on the middle slope.

**Table 7:** Two-way ANOVA results showing the interaction effects of slope position and soil depth on chemical properties of soil in the Achewa irrigated area.

Slope * Soil depth	Ca^2+^	Mg^2+^	Na^+^	K^+^	CEC	PBS (%)
	(mg/l)				(cmol (+) kg^-1^)	
Upper slope * D_1_	5.03^**e**^	2.93^**c**^	0.48^**c**^	0.26^**d**^	24.66^**c**^	35.32^**d**^
Upper slope * D_2_	7.42^**c**^	6.39^**a**^	0.73^**ab**^	0.31 cd	19.85^**d**^	74.93^**a**^
Middle slope * D_1_	5.74^**de**^	5.26^**b**^	0.39^**c**^	0.30 cd	27.28^**b**^	42.91^**c**^
Middle slope * D_2_	8.68^**b**^	7.02^**a**^	0.43^**c**^	0.41^**ab**^	24.52^**c**^	67.54^**b**^
Lower slope * D_1_	6.33^**d**^	4.93^**b**^	0.56^**bc**^	0.37^**bc**^	32.29^**a**^	37.83 cd
Lower slope * D_2_	10.76^**a**^	7.16^**a**^	0.80^**a**^	0.49^**a**^	25.63^**c**^	75.05^**a**^
CV (%)	8.15	9.42	18.61	13.54	3.20	6.26
P-value	*	*	Ns	Ns	**	**

Means values sharing the same letter in the same (column or row) do not differ statistically at the specified levels of significance. From this ** = highly significant at P < 0.01, *** = Very highly significant at P < 0.001, Ns = Non-significant at P > 0.05, CV = Coefficient of variation, LSD = Least significant difference and P = Probability, CEC = cation exchange capacity and PBS = Percent Base Saturation

Across all slope positions, surface soils contained significantly higher exchangeable cations than subsurface layers, reflecting greater organic matter accumulation, microbial activity, and cation exchange capacity in the topsoil. The exchange complex of the soils is predominantly occupied by (Ca2^+^> Mg^2+^> Na^+,^> and K^+^). This could be related to the charge density, as divalent cations (Ca and Mg) have a higher affinity towards colloidal sites than monovalent cations (Na and K). Similar orders were also reported by [38,74]; moreover, such deviations from this order can create ion-imbalance problems for plants. Considering this result, [8] also reported a similar order of abundance of basic cations on the exchangeable complex in the Middle Awash Basin, Ethiopia, and pointed out that such an order is favorable for crop production.

Based on [43] classification, the study area’s exchangeable calcium and magnesium levels are rated as medium and high, respectively, across all slope gradients.

#### 3.3.7. Cation Exchange Capacity (CEC).

**Fig 8:**
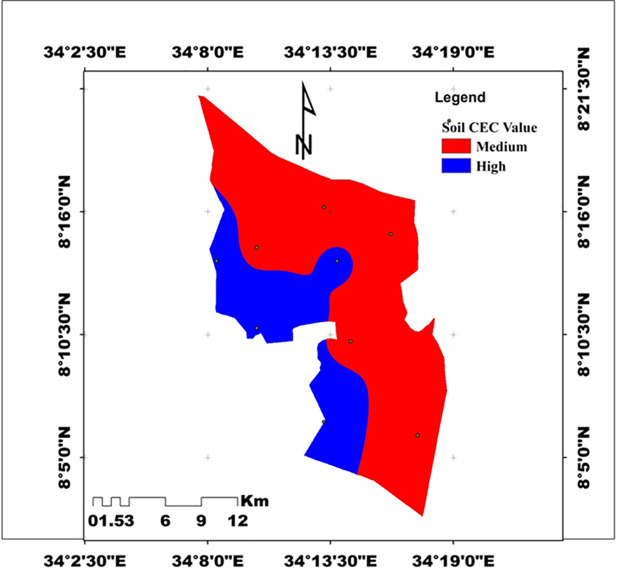
Spatial distribution of Cation exchange capacity (Source: Developed by the researchers from ArcGIS 10.4) (2024).

Cation exchange capacity (CEC) was highly significantly influenced by slope gradient, soil depth (p < 0.001), and their interaction (p < 0.01) (Tables 6–7). Across slope positions, CEC values decreased from the lower slope (28.96 cmol(+) kg ⁻ ¹) to the middle (25.90 cmol(+) kg ⁻ ¹) and upper slopes (22.26 cmol(+) kg ⁻ ¹). The higher CEC at the lower slope reflects the depositional nature of this position, where fine particles, organic matter, and leached bases accumulate, enhancing soil charge properties. In contrast, the upper slope exhibited the lowest CEC due to soil erosion, nutrient removal, and reduced organic matter input. These results agree with findings by [5,44,60], who also reported maximum CEC at lower slopes and minimum at upper slopes, largely attributed to differences in clay and organic matter distribution. According to J. R. Landon, (2014), CEC values in the study area are rated as high on the lower and middle slopes and optimum on the upper slope (Fig 8).

Interaction effects further emphasized the combined role of topography and depth. The highest CEC (32.29 cmol(+) kg ⁻ ¹) occurred in surface soils of the lower slope, while the lowest (19.85 cmol(+) kg ⁻ ¹) was recorded in subsurface soils of the upper slope (Table 6). The enrichment of CEC at the lower slope surface is attributed to the deposition of clay and organic matter, which are the main sources of negative charges that determine exchange capacity. Conversely, upper slope subsurface soils showed the lowest values due to limited residue input, intense erosion, and leaching losses of exchangeable bases. Overall, the variation in CEC across slope and depth strongly reflects the redistribution of organic matter and fine soil fractions, confirming the dominant role of organic matter over clay in controlling CEC under these conditions [49] and [70] reported that the higher mean value (26.34 cmol (+) kg^-1^) was registered under sloping lands, while the lower average value (23.49 cmol (+) kg-1) was registered under moderately steep sloping land(Fig 8).

#### 3.3.8. Percentage Base Saturation (PBS).

**Fig 9:**
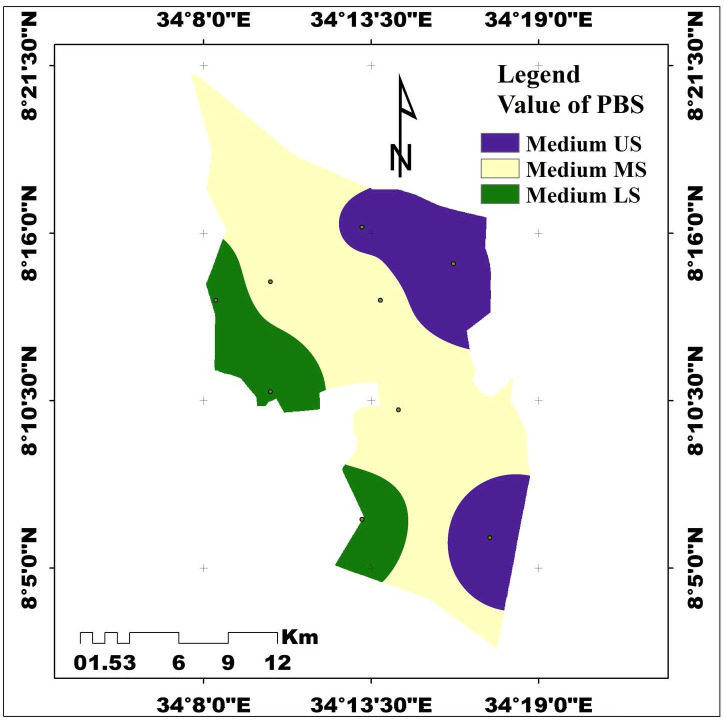
Spatial distribution of percent base saturation (Source: Developed by the researchers from ArcGIS 10.4) (2024).

Analysis of variance showed that the PBS was strongly influenced significantly (P < 0.001) by soil depth and highly significantly influenced by the interaction of slope gradients and soil depths (P < 0.01), but not significantly influenced (P > 0.05) by slope gradients. The PBS of the *Achewa* irrigated area did not show significant differences in slope gradient. However, numerically, the overall mean value of percentage base saturation among slope gradients was recorded by the following descending order: lower slope (56.44%)> middle slope (55.22%) upper slope (55.13%) (Table 6). Even though statistically it was insignificant among slope gradients, numerically the highest (56.44%) and the lowest (55.13%) percentage base saturation were found on the lower and upper slope gradients. A higher amount of PBS was recorded on the subsurface (72.50%) layer compared to the surface (38.69%) (Table 6). The result revealed that percentage-based base saturation was increasing with increasing soil depths. The maximum percentage base saturation value (72.50%) was recorded on the sub-surface soil depth, while the minimum value (38.69%) was found on the surface soil depth (0–20 cm). The primary driver for increased percentage base saturation with depth is the greater intensity of leaching and acidifying processes in the surface soil, which remove basic cations, combined with a relative accumulation or less removal of these cations in deeper, less weathered soil layers. This result is in line with the study of [54], who stated that a higher (65.23%) percentage base saturation was observed in the subsurface soil layer, while a lower (59.66%) percentage base saturation value was recorded under the surface soil layer. Paradoxically, [75] reported that higher (78.2%) and lower (77.8%) percentage base saturation values were recorded on the surface (0–20 cm) and subsurface soil depth (20–40 cm) soil depth, respectively. As per the classification set by [39], the percentage base saturation is rated high on subsurface soil depth and low on surface soil depths (Fig 9).

Considering the interaction effects, the percentage-based saturation value was significantly (P < 0.05) influenced by the interaction effects of slope gradients interacting with soil depths (Table 7). The maximum mean percentage base saturation value (75.05%) was recorded on the lower slope gradient at subsurface (20–40 cm) soil depth, while the lowest mean percentage base saturation (35.32%) was registered on the upper slope gradient of the surface soil depth (Table 6). The difference in percentage base saturation due to the interaction effect might be erosion and sedimentation, leaching and water movement, organic matter accumulation, and pH difference. By interplay of those factors results in the concentration of base cations in the subsurface soil of lower slopes, leading to a higher PBS as compared to the surface soil of the upper slope gradient. According to [39], the PBS of the study area is rated high at (20–40 cm) soil depths and low for surface layers (0–20 cm) soil depths.

#### 3.8.9. Extractable micronutrient.

Extractable micronutrients in the *Achewa* irrigated soils were significantly influenced by slope position and soil depth (Table 8). Across slope gradients, Fe and Mn varied very highly significantly (p < 0.001), Cu significantly (p < 0.05), while Zn was not affected (p > 0.05). The highest Fe (15.78 mg kg ⁻ ¹), Mn (11.08 mg kg ⁻ ¹), and Cu (2.82 mg kg ⁻ ¹) were recorded on the lower slope, whereas the lowest Mn (7.63 mg kg ⁻ ¹) and Cu (2.13 mg kg ⁻ ¹) were observed on the upper slope. Extractable Fe was lowest at the middle slope (12.37 mg kg ⁻ ¹) but did not differ statistically from the upper slope (12.97 mg kg ⁻ ¹). Although slope had no significant effect on Zn, values ranged from 1.59 mg kg ⁻ ¹ at the lower slope to 1.97 mg kg ⁻ ¹ at the middle slope. The general increase of Fe, Mn, and Cu downslope reflects erosional loss of nutrients from upper slopes and their subsequent accumulation in depositional lower slopes. These findings are consistent with findings of [45,46,50]. Based on [76], Fe and Zn levels were rated as high, while Mn and Cu were within the medium range across slope positions.

**Table 8:** Results of the two-way ANOVA showing the main effects of slope position and soil depth on micro nutrient properties in the Achewa irrigated area.

Sampling site	Extractable micro nutrient in mg kg^-1^			
	Fe	Mn	Cu	Zn
Upper slope	12.97^**b**^	7.63^**b**^	2.13^**b**^	1.94^**a**^
Middle slope	12.37^**b**^	8.23^**b**^	2.65^**a**^	1.97^**a**^
Lower slope	15.78^**a**^	11.08^**a**^	2.82^**a**^	1.59^**a**^
LSD (0.05%)	1.131	1.279	0.482	0.429
P-value	***	***	*	Ns
Soil depth in centimeters (cm)				
0_20	17.43^**a**^	10.96^**a**^	3.03^**a**^	2.15^**a**^
20_40	9.98^**b**^	7.00^**b**^	2.03^**b**^	1.51^**b**^
LSD (0.05%)	0.923	1.044	0.3939	0.350
CV (%)	6.55	11.32	15.12	18.61
P-value	***	***	***	**

Means values sharing the same letter in the same (column or row) do not differ statistically at the specified levels of significance. From this ** = highly significant at P < 0.01, *** = Very highly significant at P < 0.001, Ns = Non-significant at P > 0.05, CV = Coefficient of variation, LSD = Least significant difference and P = Probability

Soil depth exerted a stronger influence on micronutrient availability. Fe, Mn, and Cu were very highly significant (p < 0.001), and Zn was significant (p < 0.05), all showing higher concentrations in the surface (0–20 cm) than in the subsurface (20–40 cm). Surface soils contained 17.43, 10.96, 3.03, and 2.15 mg kg ⁻ ¹ of Fe, Mn, Cu, and Zn, respectively, whereas subsurface soils contained 9.98, 7.00, 2.03, and 1.51 mg kg ⁻ ¹. This vertical decline is attributed to the higher organic matter, root activity, and microbial processes in surface horizons, which are the primary sources of available micronutrients. Similar depth-related declines in micronutrients were reported by [7,64,77,78] in Ethiopian soils. Among interaction effects, only Mn was significantly affected (p < 0.01) (Table 9), with the highest concentration at the surface of lower slopes and the lowest at the subsurface of upper slopes, reflecting the combined role of erosion, deposition, and organic matter redistribution in shaping micronutrient availability.

**Table 9:** Two-way ANOVA results showing the interaction effects of slope position and soil depth on micro nutrient properties of soil in the Achewa irrigated area.

Slope * Soil depth	Extractable micro nutrient in mg kg^-1^			
	Fe	Mn	Cu	Zn
Upper slope * D_1_	16.28^**b**^	8.32^**bc**^	2.42^**b**^	2.09^**a**^
Upper slope * D_2_	9.66^**d**^	6.93 cd	1.83^**c**^	1.78^**a**^
Middle slope * D_1_	15.71^**b**^	10.31^**b**^	3.09^**a**^	2.17^**a**^
Middle slope * D_2_	9.03^**d**^	6.14 cd	2.20^**bc**^	1.76^**a**^
Lower slope * D_1_	20.31^**a**^	14.25^**a**^	3.57^a^	2.18^**a**^
Lower slope * D_2_	11.26^**c**^	7.91 cd	2.07^**bc**^	1.00^**b**^
CV (%)	6.55	11.32	15.12	18.61
P-value	Ns	**	Ns	Ns

Means values sharing the same letter in the same (column or row) do not differ statistically at the specified levels of significance. From this ** = highly significant at P < 0.01, *** = Very highly significant at P < 0.001, Ns = Non-significant at P > 0.05, CV = Coefficient of variation, LSD = Least significant difference and P = Probability

### 3.4. Pearson correlation analysis

The Pearson correlation matrix revealed strong interrelationships among soil physical and chemical properties. Sand showed a strong negative correlation with clay (r = –0.919), reflecting the natural textural trade-off, while bulk density (Bd) was positively associated with silt (r = 0.580) but strongly and negatively correlated with total porosity (Tp, r = –1.000), organic matter (OM, r = –0.748), organic carbon (OC, r = –0.749), and total nitrogen (TN, r = –0.729), indicating that organic-rich soils are less compacted (Table 10). Conversely, Tp was positively correlated to OM, OC, TN, and especially available phosphorus (Avp, r = 0.896), suggesting enhanced nutrient availability in well-aerated soils. Soil pH correlated positively with clay (r = 0.531), OM (r = 0.495), and Mg (r = 0.586), implying that finer-textured, organic-rich soils buffer acidity more effectively. Cation exchange capacity (CEC) showed very strong positive correlations with OM and OC (r = 0.846 each) and Avp (r = 0.808), underscoring the critical role of organic matter in nutrient retention. Exchangeable bases were also highly interlinked, with strong associations between Ca, Mg, and K (Ca, Mg r = 0.790; Ca–K r = 0.836; Mg–K r = 0.712). Percent base saturation (PBS) correlated strongly with Ca (r = 0.821), Mg (r = 0.869), Na (r = 0.593), and K (r = 0.559), demonstrating that exchangeable cations largely determine soil base saturation status. Overall, organic matter emerged as the major driver of soil fertility by enhancing CEC, nutrient availability, and structural stability.

**Table 10:** Interaction effects of slope and soil depth on extractable micro nutrient of soil in Achewa irrigated area.

	Sand	Silt	Clay	BD	TP	pH	EC	OM	OC	TN	AVP	Ca	Mg	Na	K	PBS	Fe
Sand	1.00																
Silt	0.03	1.00															
Clay	0.91*	−0.42	1.00														
BD	0.33	0.58*	−0.53*	1.00	−1.00												
TP	−0.33	−0.58*	0.53*	−1.00	1.00												
pH	−0.28	−0.70*	0.53*	−0.45	0.45	1.00											
EC	−0.10	−0.21	0.18	−0.36	0.36	0.40	1.00										
OM	−0.21	−0.49*	0.38	−0.75*	0.75	0.50*	0.58*	1.00									
OC	−0.21	−0.49*	0.38	−0.75*	0.75*	0.50*	0.58*	1.00	1.00								
TN	−0.11	−0.10	0.14	−0.73*	0.73*	0.15	0.43	0.73*	0.73	1.00							
AVP	−0.44	−0.64*	0.65*	−0.90*	0.90*	0.57*	0.44	0.73*	0.73	0.65*	1.00						
Ca	0.52*	−0.18	0.54*	0.15	−0.15	0.47*	−0.07	−0.29	−0.29	−0.50*	0.09	1.00					
Mg	−0.17	−0.40	0.31	0.22	−0.22	0.59*	0.17	−0.18	−0.18	−0.53*	0.01	0.79	1.00				
Na	0.56*	0.07	0.48*	0.04	−0.04	0.01	0.03	−0.32	−0.32	−0.26	0.15	0.54	0.42	1.00			
K	0.50*	−0.39	0.60*	−0.18	0.18	0.61*	0.11	0.07	0.07	−0.13	0.38	0.84	0.71	0.36	1.00		
PBS	−0.21	−0.05	0.21	0.50*	0.50*	0.21	−0.13	0.56*	0.56*	−0.78*	−0.31	0.82	0.87	0.59	0.56	1.00	
Fe	−0.12	−0.16	0.17	-.0.74	0.74*	0.02	0.31	0.77	0.77*	0.89*	0.62	−0.66	−0.66	−0.28	−0.33	0.88	1.00

**and * correlation is significant at p < 0.01 level and p < 0.05 level (2-tailed) respectively

### 3.5. Principal component analysis (PCA)

Principal Component Analysis (PCA) was employed to classify soil parameters from samples collected across three slope locations (upper, middle, and lower) and two depths (0–20 cm and 20–40 cm), elucidating the variance captured by a limited set of variables [79]. Before conducting the PCA, the Kaiser-Meyer-Olkin (KMO) test confirmed an acceptable sampling size with a value greater than 0.05, while Bartlett’s Sphericity test assessed the strength of association among variables at a 0.05 significance level [80]. Eigenvalues greater than one were used for additional analysis [81]. In our study, the KMO measure of sample size adequacy was 0.752, and the Bartlett test of Sphericity yielded a value less than 0.05. Two principal components (PCs) with eigenvalues greater than one were selected for further analysis, while others were excluded.

**Fig 10:**
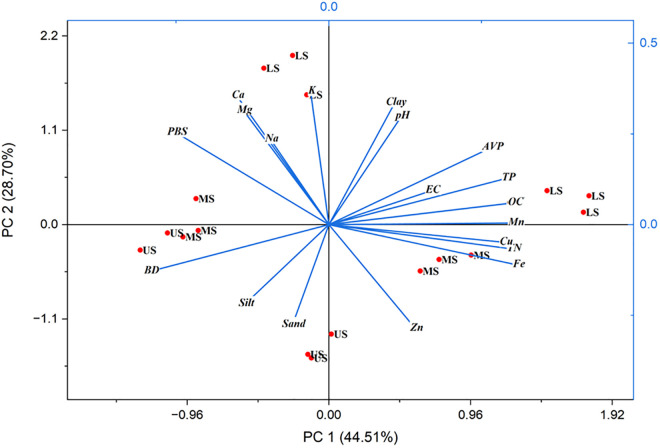
Bi-plot of the two PCA (Source: Developed from laboratory data (2024).

In the *Achwa* small-scale irrigation study, PCA 1 explained 44.51% of the total variance, showing strong positive associations with clay, organic matter, and nutrient levels, alongside negative correlations with sand and bulk density. PCA 2 accounted for an additional 28.70% of the variance, highlighting a positive relationship with silt (Table 11) (Fig 10). Collectively, these two principal components explained over 73% of the total variability in soil attributes, underscoring their significance in understanding soil dynamics. The communality estimates revealed that factors such as organic matter and clay contributed over 90% to the variation, while bulk density, pH, and nutrient levels also accounted for significant portions. This analysis emphasizes the importance of specific soil parameters in management practices, particularly in optimizing soil health and addressing slope stability issues.

**Table 11:** Principal component analysis of soil parameters.

Soil Parameters	PC1	PC2	Communality Estimates
Sand (%)	−0.0597	−0.1370	0.638
Silt (%)	0.1081	0.3359	0.645
Clay (%)	−0.2923	−0.1283	0.912
BD (g cm ⁻ ³)	0.2923	0.1282	0.813
TP (%)	0.1188	0.2985	0.547
pH	0.1682	0.0938	0.439
EC	0.3035	0.0603	0.551
OM (%)	0.3036	0.0604	0.645
OC (%)	0.3014	−0.0670	0.612
TN (%)	0.2662	0.2125	0.569
AVP	−0.1507	0.3550	0.721
Ca² ⁺ (cmol (+) kg ⁻ ¹)	−0.1399	0.3144	0.851
Mg²⁺ (cmol (+) kg ⁻ ¹)	−0.0956	0.2351	0.878
Na⁺ (cmol (+) kg ⁻ ¹)	−0.0299	0.3670	0.582
K⁺ (cmol (+) kg ⁻ ¹)	−0.2503	0.2534	0.690
Fe	0.3089	−0.1109	0.604
Mn	0.3049	0.0047	0.597
Cu	0.2885	−0.0489	0.59
Zn	0.1417	−0.2863	0.562
Eigenvalue	8.90	5.40	
Variability	44.5	28.70	
Cumulative Variance (%)	44.50	73.21	

Considering slope position and soil depth is critical, as soil properties naturally vary across landscapes due to gravitational forces, erosion, and deposition processes [82]. Soils at the top of slopes are often characterized by lower organic matter content and increased erosion, while those at the bottom tend to accumulate finer particles and organic material. The observed negative correlation between sand and PCA 1 suggests that soils at the top of the slope may exhibit higher sand content and lower overall fertility, consistent with findings by [13], who noted shallower soils with low nutrient and water availability on steeper slopes. Conversely, the positive association of clay with PCA 1 implies that lower slope positions may benefit from increased water retention and nutrient availability.

### 3.6. Implications for sustainable socio-economic implications of soil fertility management and environmental conservation

The study revealed that soil fertility in the *Achewa* irrigation area is strongly influenced by slope gradient and soil depth, with lower slopes showing higher nutrient retention (OM, TN, CEC, and exchangeable bases) compared to upper slopes, which are more vulnerable to erosion and nutrient depletion. This spatial variability has clear socio-economic implications, as farmers cultivating upper slopes face lower productivity and higher input costs to compensate for nutrient loss. In contrast, farmers on lower slopes may achieve better yields but risk soil compaction and drainage issues if management practices are not improved. To enhance agricultural sustainability and farmer livelihoods, slope-specific interventions are essential. On upper slopes, soil and water conservation measures such as terracing, mulching, and agroforestry can mitigate erosion and preserve soil fertility [45,56]. On lower slopes, integrated nutrient management combining organic amendments and judicious fertilizer application will sustain productivity without causing nutrient leaching or waterlogging [3]. Policymakers and extension services should prioritize training farmers on topography-sensitive land use planning and promote soil fertility management for site-specific input use. Such practices can reduce input costs, improve yields, and strengthen household food security while minimizing long-term land degradation and environmental risks.

## 4. Conclusions and recommendations

### 4.1. Conclusion

This study was conducted to analyze the effect of slope gradient and soil depth on selected soil properties, exchangeable bases, and extractable micro nutrients of *Achewa* irrigated land, Itang special District in the Gambella region, southwest of Addis Ababa, with an average altitude of 424 meters above sea level. The research result revealed that selected soil parameters, exchangeable bases, and extractable micro nutrients were significantly affected by different levels of slope gradients, soil depth, and, to some extent, by their interactions. Based on slope gradients, clay content, TP, pH, OM, TN, and Av. P, exchangeable bases (Ca, Na, K), CEC, and extractable micro nutrients (Fe, Mn, and Cu) were higher on the lower slope gradients, while sand fraction, BD, and extractable Zn were maximum on the upper slope positions. Moreover, in account of soil depth, sand fraction, TP, OM, TN, Av. P, CEC, and available micro nutrients (Fe, Mn, Cu, and Zn) were higher in surface (0–20 cm) soil depth, meaning they decreased with increasing soil depth. In contrast, clay content, BD, soil pH, and exchangeable bases (Ca, Mg, Na, and K) values increased with increasing soil depth; they had a direct relationship with soil depth. In addition to the main effects, slope gradient interacts with soil depth had significantly affects selected soil properties, to some extent, on exchangeable bases and extractable micro nutrients. Highest values of TP, pH, OM, TN, AV.P, and CEC were recorded on the lower slope gradient on the surface (0–20 cm) soil depth. Additionally, clay content, exchangeable Ca, Mg, and PBS value were highest on the lower slope gradient under subsurface (20–40 cm) soil depth.

### 4.2. Recommendations

From the result of this study, the following recommendations are suggested:-

➢ Soil and Water Conservation: Introduce biological (e.g., agroforestry, grass strip) and physical (e.g., terraces, bunds) soil and water conservation in the upper slopes, where soil loss and nutrient leaching are serious. Encourage conservation tillage and mulching so that organic matter and soil structure are maintained.
➢ Encourage participatory land management strategies that integrate local farmers’ practices with scientific recommendations to ensure sustainable use of soils in the *Achewa* irrigation area.
➢ Extend soil mapping to other micro-catchments in Gambella so as to create larger datasets in land use planning.
➢ Sustainable land use and nutrient management practices should be introduced to enhance agricultural productivity in the region.

## Supporting information

S1 File Soil laboratory Results(Row data) of Achewa small-scale irrigation site. (XLSX)
